# Membrane-elution analysis of content of cyclins A, B1, and E during the unperturbed mammalian cell cycle

**DOI:** 10.1186/1747-1028-2-28

**Published:** 2007-09-24

**Authors:** Stephen Cooper, Michelle Paulsen, Mats Ljungman, Dang Vu-Phan, Duyang Kim, Mariam Gonzalez-Hernandez

**Affiliations:** 1 Department of Microbiology and Immunology, University of Michigan Medical School, Ann Arbor, Michigan 48109-0620, USA; 2 Department of Radiation Oncology, University of Michigan Medical School, Ann Arbor, Michigan 48109-2200, USA

## Abstract

**Background:**

Problems with whole-culture synchronization methods for the study of the cell cycle have led to the need for an analysis of protein content during the cell cycle of cells that have not been starved or inhibited. The membrane-elution method is a method that allows the study of the cell cycle by producing a culture of unperturbed, synchronized cells.

**Results:**

The Helmstetter membrane-elution method for the continuous production of newborn, unperturbed, mammalian cells has been enhanced so that the collection of cells of different cell cycle ages is automated, reproducible, and relatively inexpensive. We have applied the automated membrane-elution method to the analysis of cyclin content during the cell cycle. Cyclin E protein was invariant during the cell cycle. Cyclins B1 and A accumulated continuously during the cell cycle and were degraded at mitosis. Newborn cells had ~0.5% of the cyclin B1 content of dividing cells.

**Conclusion:**

The expression patterns of cyclins A, B1, and E can be explained by constant mRNA levels during the cell cycle. Previously reported phase specific variations of the cyclins are not strictly necessary for cell-cycle progression. Cells produced by membrane-elution are available to other laboratories for analysis of the cell cycle.

## Background

We describe an automated approach that easily yields cells in all phases of the mammalian cell cycle. Further, we apply this method to the measurement of cyclin contents during the normal mammalian cell cycle. The automated membrane-elution method (colloquially referred to as the "baby machine") allows numerous replications of cell-cycle analyses without long-term, labor-intensive, continuous sampling of cells. The automated "baby machine" method described here produces cells without any growth inhibitory treatments or changes in growth conditions. The automated method produces cells that pass normally and unperturbed through the mammalian cell cycle.

We use the term "unperturbed" in a very limited way to distinguish the cells studied here from cells that have been treated with inhibitors or subjected to starvations or media limitations that arrest cell growth prior to allowing growth to resume. Cells subjected to starvation or inhibition are considered "perturbed" cells, in contrast to the cells studied here that are maintained in steady-state conditions throughout the experimental manipulations. The initial description of the mammalian membrane elution method similarly tried to distinguish the cells produced by this method from those using starvation or inhibition. The term used was "minimally disturbed" [1] to emphasize that the cells produced by membrane-elution were more normal and unperturbed compared to cells subjected to long periods of inhibition or starvation.

Automation of the membrane-elution method has the benefits of reliability and reproducibility, without requiring manual collection of cells over many hours. This method thus addresses two major problems in cell-cycle analysis.

One problem stems from the relatively long division cycle of eukaryotic cells. Obtaining cells of different cell-cycle ages from a starting synchronized culture requires long hours of collection with many inconvenient collection times.

Another problem arises from artifacts that may be introduced by commonly used whole-culture synchronization methods. Most treatments proposed to induce synchrony in a culture involve starvation or inhibition. These treatments may induce periodicities or variations that may not exist in the normal, unperturbed cell. While it is never possible to eliminate all effects of experimental manipulations, no matter how gentle, we emphasize the use of the term unperturbed to distinguish the cells produced by the automated membrane-elution method from synchronization methods that involve long-term inhibition of growth by starvation or chemical treatments.

**Figure 1:**
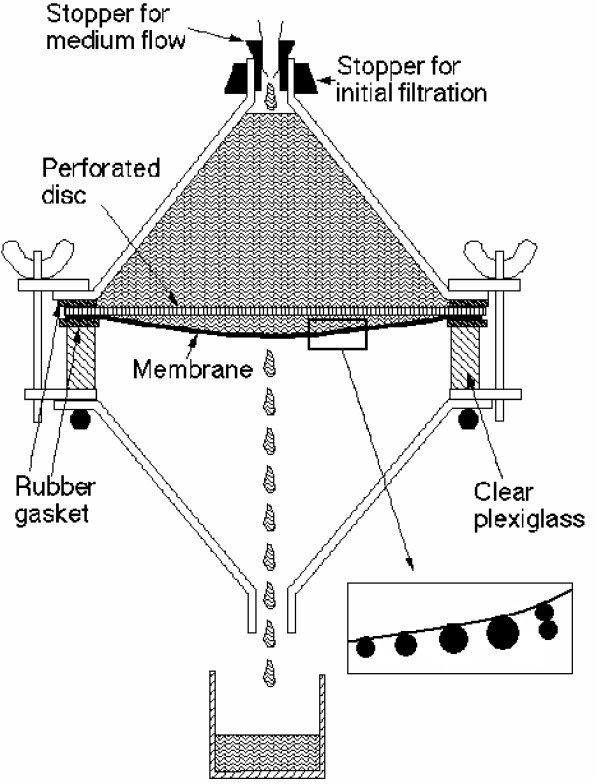
Diagram of membrane-elution apparatus. The apparatus is inverted at the start of an experiment, with cells collected on the membrane surface by suction. Then the apparatus is inverted, and medium fills the space above the membrane as shown. Cells grow on the membrane surface. Newborn cells are released at division, with one cell entering the eluate and the sister cell remaining bound to the membrane.

The bacterial membrane-elution method, the "baby-machine", was developed by Helmstetter over four decades ago [2,3]. Bacteria were bound to a membrane, the membrane was inverted, and fresh medium was pumped through the membrane. (A diagram of the apparatus is shown in Fig. 1.) Only newborn cells produced by division of the bound cells were released from the membrane. Because cells of all cell-cycle ages were originally bound to the membrane, and because only one of the two newborn daughter cells was released to the eluate, newborn cells were released continuously over many generations. The bacterial membrane-elution method led to the elucidation of the pattern of DNA replication during the division cycle of bacteria [4-13] as well as numerous other synthetic patterns during the bacterial cell cycle [14-19].

The membrane-elution method has been adapted for eukaryotic cells by Helmstetter and his colleagues [20,1,22]. The eukaryotic membrane-elution method, like the bacterial system, produces newborn cells continuously from cells bound to a membrane. We have developed a simple enhancement of the Helmstetter method that allows cells to be automatically collected and grown to different cell-cycle ages. This automated method now allows simple and efficient production of cells in all phases of the cell cycle.

An example of how the study of unperturbed cells can yield the normal pattern of gene expression and modification during the cell cycle comes from a study of retinoblastoma (Rb) protein phosphorylation. Previous work from this laboratory reexamined the view that Rb protein is phosphorylated in a cell-cycle specific manner, specifically in the G1-phase of the cell cycle. It was shown that when cells are grown without any inhibitory signals, all of the Rb protein is phosphorylated [23] with no cell-cycle specific (i.e., no G1-phase specific) phosphorylation observed [24]. When cells are grown with some cells having inhibited growth (as may arise during microcolony growth by adherent cells on a flat surface), one can get results that lead to the conclusion that there is cell-cycle specific Rb phosphorylation [24].

We have now applied the automated membrane-elution method to the analysis of cyclin content during the division cycle. Cyclins A and B1 are low in newborn cells and increase continuously during the cell cycle. Degradation of cyclins A and B1 occurs at the end of the cell cycle. Cyclin E content is invariant during the cell cycle.

## Results

### Membrane elution analysis

By collecting newborn cells sequentially in separate fractions in a warm room one can obtain, after overnight collection, a series of fractions where the cells have grown for different lengths of time. This eliminates the need to collect cells manually during the entire period of growth during the cell cycle. The automated membrane-elution technique is described in detail in the Methods section below.

**Figure 2:**
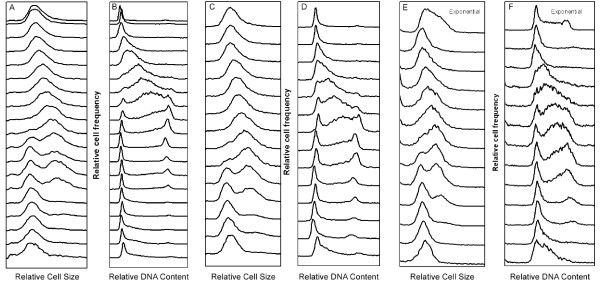
Changes in the size distribution and DNA content of cells during the cell cycle. Three independent experiments are presented (A-B, C-D, E-F). Each line is separated in time by one hour in graphs A-D, and by 1.33 hours in graphs E-F. (In graphs A and B the first two lines are separated by fifteen minutes with subsequent lines separated by one hour.) Newborn cells at the top are from the last fraction collected. Each subsequent line represents cells that have grown for one hour longer than the previous line (or 1.33 hours in graphs E and F.) The size distributions A, C, and E correspond with the respective DNA distributions B, D, and F. Exponential size and DNA distributions are shown at the top of panels E and F. The results shown in A-D are for 10% CCS while the panels E and F used 5% CCS.

The size distributions and DNA contents for three typical, independent experiments are shown in Fig. 2. The newborn cells increased in size and went through a relatively short period of cell division to produce newborn cells (panels A, C, E). Concurrently, the same population of newborn cells had a G1-phase amount of DNA in newborn cells, then an S-phase amount, and finally a G2-phase amount of DNA (panels B, D, F). After division, small cells with a G1-phase amount of DNA were again obtained. The results from these three experiments are representative of many (over 30 at this time) experiments. These results show the reproducibility of the automated membrane-elution method in obtaining populations of cells of different ages during the cell cycle.

### Analysis of cyclins A, B1, and E content during the cell cycle

**Figure 3:**
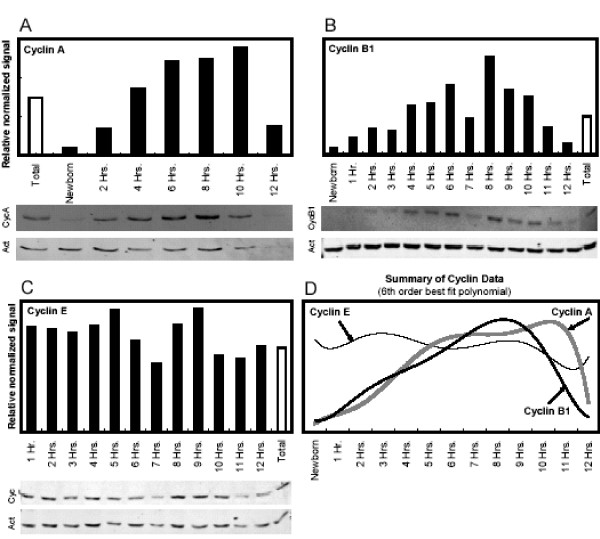
Cyclin content during the cell cycle. Extracts from cells of different ages were separated by SDS-PAGE. Cyclin contents were measured by immunoblotting (black bars). All protein signals were normalized to actin signal in the same lane. Each blot also included an extract from an exponentially growing culture, representing cells of all ages (open bars). This total sample is expected to yield an average cyclin content; in the three experiments shown here, this expectation is fulfilled. Starting from the upper right and proceeding counterclockwise, the three panels show cyclins B1, A, E, protein levels during the cell cycle. In the lower right, the best fit 6^th ^order polynomial trendline for the three experiments are shown.

Cyclin B1 content during the division cycle is shown in Fig. 3B. Cyclin B1 content was low in newborn cells, increased steadily as the cells progressed through the cell cycle, and declined dramatically coincident with division. This produced a population of newborn cells with little cyclin B1. A summary of the cyclin B1 data in the form of a 6th order best-fit polynomial (Excel) is shown in Fig. 3D.

Because of the design of the experiment in Fig. 3B, the cyclin B1 content in the "newborn" cells was determined on cells that were 15–60 minutes post-division (see Methods). In order to determine the degree of cyclin B1 loss at mitosis, the cyclin content of cells 0–15 minutes old was determined. Protein from exponentially growing cells and newborn cells (0–15 minutes old) were loaded on a Western blot such that there was 100 times as much newborn cell protein (70 μg) as exponential cell protein (0.7 μg). There was a small signal from the newborn protein but a stronger signal from the protein from exponentially growing cells. The cyclin B1 signal in the newborn cells is, therefore, on a protein weight basis, less than 1% of the exponential cell protein signal. As the exponential protein signal is the result of averaging the cyclin B1 over the entire cell cycle, including newborn cells, the cells at the end of the cycle have more cyclin B1 signal compared to the average signal. We conclude that cyclin B1 decreased by approximately a factor of 200 at mitosis.

The finding of negligible cyclin B1 in newborn cells has implications for understanding the efficiency of membrane-elution and the purity of the eluted cells. The very low cyclin B1 content indicated that there is essentially no random loss of cells from the membrane; essentially all eluted cells were the result of cell division. If the newborn cells actually had absolutely no cyclin B1, this would set a limit to contamination by random elution of cells from the membrane to approximately 0.5%. If, however, the newborn cells had 0.5% residual cyclin B1, then one could conclude that all of the eluted cells arise by division with no contamination by random cell loss from the membrane.

Cyclin A content during the division cycle had a pattern similar to that of cyclin B1 (Fig. 3A). The cyclin A and B1 patterns are compared with best-fit trendlines in Fig. 3D. We suggest that cyclins A and B1 protein increase continuously during the cell cycle, with rapid degradation of these cyclins at the end of the cell cycle during mitosis and division. There is no indication in our analyses of any variation in the rates of increase of cyclins A and B1 protein during the cell cycle prior to division. That is, the increase in cyclins A and B1 are continuous and essentially linear during the interphase portion of the cell cycle.

Cyclin E appears invariant during the cell cycle (Fig. 3C); the data are summarized in Fig. 3D. This result is somewhat different from the current view of cyclin E variation during the cell cycle (for example, see [25]).

## Discussion

### Summary of cyclin content during the cell cycle

**Figure 4:**
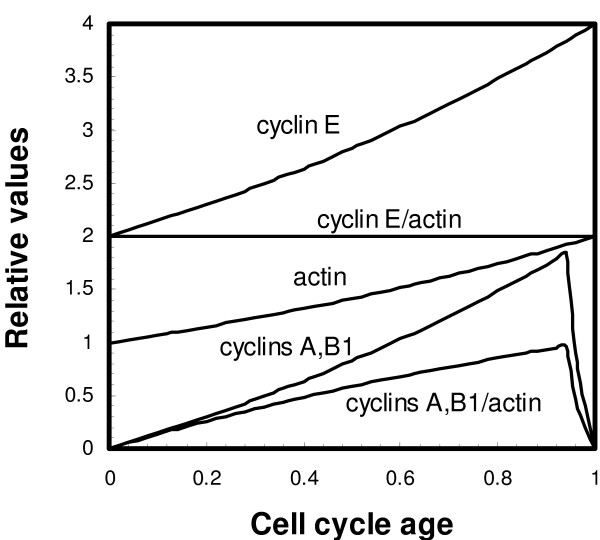
Summary of proposed cyclin patterns during the cell cycle. Both cyclin E and actin are proposed to increase exponentially during the cell cycle, and the ratio is a constant indicated by the horizontal line (cyclin E/actin). Cyclins A and B1 increase during most of the cell cycle (formula is 2(2^x^-1) where x is the cell age), with a decrease in cyclin content at the end of the cell cycle. The ratio of cyclins A and B1 to actin (cyclins A, B1/actin) is shown, and is similar to the pattern for absolute cyclin A, B1 content.

A summary of the patterns of cyclin content during the division cycle is presented in Fig. 4. Cyclin E and actin are synthesized exponentially during the division cycle; normalization of cyclin E content to actin gives a constant ratio. Cyclins A and B1 increase continuously, with no changes in the rate of increase, from low values in newborn cells to a peak near the end of the cell cycle. There are decreases in cyclins A and B1 as cells undergo mitosis and cytokinesis. The absolute and normalized (to actin) patterns of cyclins B1 and A are shown in Fig. 4.

### Cyclin E expression during the cell cycle

Our results show an invariant cyclin E protein content during the division cycle (Fig. 3D). This result is different from the current view of cyclin E content during the mammalian cell cycle. For example, in a review of cyclin E content during the cell cycle, Moroy and Geisen [26] stated "E-type cyclins (cyclin E1 and cyclin E2) are expressed during the late G1 phase of the cell cycle until the end of the S-phase...," and "Expression of cyclin E varies during the cell cycle with peaks at the G1-S phase boundary..." [26]. Among the experiments cited in support of the conclusion of cycle-specific variation in cyclin E content are single cell assays on cells whose age is determined by time-lapse videography [27] and serum starvation/refeeding to analyze cyclin E mRNA synthesis [28]. The cyclin E protein has been assayed by in situ single-cell assay and by activation of kinase activity as a measure of cyclin E activity.

These published cyclin E results should be contrasted to the results presented here, where we assay the cyclin E on Western blots, using cells passing unperturbed through the cell cycle. The constant amount of cyclin E during the division cycle (relative to actin) implies that cyclin E is made exponentially during the division cycle in parallel with an exponential increase [29] in cell mass during the division cycle. This result implies that cyclical variation of cyclin E content may not be absolutely necessary for passage through the cell cycle.

### Cyclins A and B1 content during the cell cycle

The patterns of cyclins A and B1 content during the division cycle (Figs. 3D and 4) show a continuous, essentially linear increase during the majority of the cell cycle with a sharp decrease in cyclin content at division. We consider the patterns of cyclins A and B1 to be essentially identical (although slight differences are apparent). The approximately linear increases of cyclins A and B1 during the cell cycle may indicate invariant gene expression (i.e., mRNA content) during the cell cycle for these cyclins. During most of the cell cycle (before mitosis or cell division commences) a constant amount of mRNA would lead to a continuous and approximately linear increase in cyclin content relative to total protein (as monitored by actin content).

### Automation of the membrane-elution method

The automated membrane-elution method allows the production of cells of different cell-cycle ages automatically and easily. The cells fit the synchronization criteria for unperturbed cells passing through the normal cell cycle.

Rather than manually collecting cells, newborn cells are collected sequentially in a fraction collector. By allowing the cells in different fractions to grow for various lengths of time until a common collection time one has cells in each vial representative of a specific cell cycle age. By making cell-cycle analysis simple, reproducible, and not hostage to collecting cells at problematic time points (e.g., 3:00 am), one can perform many experiments and obtain reproducible and consistent results.

The appearance of small number of residual cells with a G1-phase amount of DNA after periods of cell growth may be due to some cells, upon occasion, not starting DNA replication along with the original cohort of cells, or those cells with a G1-phase amount of DNA may be the result of some cells moving rapidly through the cell cycle to divide and produce cells with a G1-phase amount of DNA. A small amount of contamination with adventitiously released cells that then divide could also account for these cells. The retention of such cells with a G1-phase amount of DNA has also been noted when flasks (rather than vials) have been used to collect cells from membrane-elution [30], suggesting that the appearance of these cells may not be a result of our modification of the Helmstetter method.

### Modifications of the original mammalian membrane-elution method

It is important to distinguish the original membrane-elution method as practiced in the Helmstetter laboratory [20,1,22] with the automated method described here. One difference is the use of glass vials to collect newborn cells rather than plastic flasks as used by the Helmstetter Laboratory. It is possible that the plastic flasks have a much flatter surface than the glass vials, and this may lead to minor variations in growth and division of the newborn cells.

Also, the substitution of Cosmic Calf Serum for Fetal Bovine Serum may affect the growth rate, but it is hard to imagine that the fundamental pattern of cell growth would be affected by this change in medium components. It should not be missed that the cost of Cosmic Calf Serum is approximately 20% that of Fetal Bovine Serum. As large volumes of media are required for the membrane-elution method, this modification leads to a much more economical approach to cell-cycle analysis.

The collection of cells in flasks also allowed the cells to be incubated in a sealed environment. In contrast, the collection of cells in glass vials led to the incubation of cells in open vials. This could lead to a small amount of evaporative cooling, particularly in the vials at the start of collection. This would lead to some stretching out of the second cell cycle, although we find that the first cell cycle during a membrane-elution experiment has a normal interdivision time.

Newborn cells eluted immediately after placement of the cells on the membrane are slightly smaller than newborn cells eluted after a long period of elution. This phenomenon has also been observed by the Helmstetter laboratory [30]. Yet these cells that are produced by long term-elution are normal and in steady-state growth [30]. While the newborn cells produced by long-term elution are larger, and may have a shorter G1 phase as a result, they pass normally through the division cycle without any indication that the cell-cycle passage is altered in these experiments.

The most important modification or enhancement of the original Helmstetter method is the automation of cell collection. This allows the collection of newborn cells and the production of cells in all phases of the cell cycle without labor-intensive continuous sampling from an initial synchronized culture. The automated method is also very reproducible. The convenience offered by the automated method will allow many repetitions of experiments on measurements of biosyntheses during the cell cycle.

### Availability of membrane-eluted cells for the scientific community

The cells produced by the automated membrane-elution method described here are available to other laboratories for collaboration on various aspects of cell-cycle study. Laboratories that are currently analyzing proteins that appear relevant to cell-cycle progression can now check the pattern of synthesis on cells produced by membrane elution. Please write cooper@umich.edu to discuss possible collaborative efforts.

## Methods

### Cells

L1210 cells, a mouse leukemic line (ATCC designation CCL219) were used for all experiments. These cells are non-adherent and grow with a doubling time of approximately 9–11 hours.

### Media

Liebovitz's L-15 medium (cellgro by Mediatech, Herndon, VA 20171) was supplemented with 2 mg/ml glucose, 100 U/ml penicillin, 100 μg/ml streptomycin, and 10% Cosmic Calf Serum (CCS). Cosmic Calf Serum (Hyclone, Inc.) is a modified calf serum that substitutes for Fetal Bovine serum (FBS). The cost of CCS is between one-fifth and one-tenth that of FBS. One can go as low as 5% CCS and get essentially the same growth rate as 10% CCS, thus making the method even more economical. The buffering in L-15 medium allows cell growth and pH maintenance without a CO_2 _atmosphere. Cells were grown at 37°C in sealed flasks prior to a membrane-elution experiment. Cells were kept below 200,000 cells/ml during exponential growth. The CCS was filtered (0.22 or 0.45 micron pore filters) before a membrane-elution experiment to avoid clogging the membrane.

### The membrane holder

The membrane holder apparatus has been described previously [1,20,22], but some of the details will be presented here. A support screen (Millipore, catalogue number YY3014234) was secured in a holder with rubber gaskets so that a membrane (Millipore catalogue number GSWP14250; 142 mm nitrocellulose membrane, 0.22 micron pores (identical results are obtained with Millipore HAWP14250 which is a nitrocellulose membrane with 0.45 micron pores)) lay directly on the support screen. A Lucite ring confined the liquid to the top of the membrane. Rubber gaskets between the membrane and Lucite ring prevented leakage. The support screen lay over a funnel that can be inserted into a side-arm flask to allow suction to pull the medium through the membrane (Fig. 1).

### The automated membrane-elution method

Cells were grown to a concentration of less than 200,000 cells per ml to obtain approximately 60–70 million cells. For example, 600 ml of cells at 100,000 cells per ml gave 60 million cells. All experiments were carried out in a warm room (37°C) with warm media and buffers. The membrane holder and medium reservoir were kept in a full-view incubator within the warm room to ensure constant temperature. To start the production of newborn cells, 50 mls of PBS (phosphate-buffered saline) with 10 μg/ml concanavalin A was filtered through the membrane. Upon completion of the filtration no residual liquid remained. PBS (100 ml) was then filtered through the membrane to remove unbound concanavalin A; again no residual liquid remained. Cells in 300–600 mls of medium were filtered slowly onto the membrane with gentle suction over 3–5 minutes. When approximately 20–30 mls of liquid remained above the membrane, the liquid was poured off so that the cells were never dried and exposed to air. The membrane apparatus was inverted and filled with fresh medium. Medium from a 4 L reservoir was pumped through the membrane at a rate of 2.0–3.0 ml/min. After approximately 30 minutes, the unbound and weakly bound cells had been removed. The unbound cells obtained from this initial flow of medium through the membrane were collectively referred to as the "wash-off". The wash-off was usually between 10–20% of the input cells. Thus, 80%–90% of the initial cells were bound to the membrane. The eluted cells were monitored until it was determined that only newborn cells were eluted as determined by cell size. Then the membrane was placed over a large funnel connected by tubing to a peristaltic pump. The pump connected to the bottom of the funnel pumped liquid at approximately 4.0–10.0 ml/min into the vials within the fraction collector. This prevented pooling of cells in the funnel. The eluate from the membrane was collected in sterile glass vials (40 ml) in a Pharmacia fraction collector. Although the entire system (medium reservoir, pumps, membrane holder, fraction collector) was in a warm room, an incubator box was built around the fraction collector. The incubator box contained a thermocouple-controlled heater with a fan to maintain a constant temperature. Thus, even when the warm room door was occasionally opened, there was no change in the temperature of the collected cells.

Fractions were collected for 15-minute or 20-minute intervals yielding 35–40 ml of media with newborn cells. The concentrations of cells in each vial were generally less than 25,000/ml; no inhibition of growth occurs at this low cell concentration. Since cells in each of the vials in the fraction collector grew for different lengths of time prior to cell harvesting, each vial contained cells at different cell cycle ages. At the end of a collection period (15–22 hr), the vials were removed to an ice bath. The cells were collected by centrifugation for analysis of the cell size distribution, cellular DNA content, and protein content. In a typical experiment, the first vial of each group of four or five vials was used for cell sizing and assay of DNA contents and the remaining three or four vials were pooled for protein analysis by western blotting. Because of the reduced need for continuous collection of cells it is possible to do two complete membrane-elution experiments a week.

### Cell counting and cell sizing

Cells were counted and sized in L-15 medium using a Beckman/Coulter Z2 Particle Counter and Size Analyzer. The data were collected and analyzed using the Z2 AccuComp program from Beckman Coulter (version 3.01). Size distributions were plotted by exporting the AccuComp data to an Excel spreadsheet program.

### DNA analysis

Cells were collected by centrifugation, washed with cold phosphate buffered saline (PBS, pH 7.4), and resuspended in 70% ethanol. Cells in 70% ethanol were collected by centrifugation, the ethanol removed, and the cells were taken up in PBS containing propidium iodide (50 μg/ml) and RNAse A (100 μg/ml). The cells were analyzed in a Becton-Dickinson Cantu FACS analyzer. Further analysis of the flow cytometry data was performed with WINMdi software.

### Western blotting

Cells from a membrane-elution experiment or from exponential growth (total cells) were collected by centrifugation and washed once with cold PBS. The cells were collected by centrifugation in a microfuge tube and freeze-dried. The dried cell pellets were lysed with NP40 lysis buffer. Equal amounts of protein were loaded with SDS and 2-mercaptoethanol loading buffer and separated on 10% polyacrylamide gels. After transfer to a low fluorescent background PVDF-FL membrane (Millipore) specific protein contents were determined by Western blotting with primary antibodies against actin (Sigma) and cyclins A, B1, and E (Santa Cruz) and IR dye conjugated secondary antibodies from Li-COR. The blots were scanned with a Li-COR scanner at 680 nm (actin) and 800 nm (cyclins) wavelengths. The results were analyzed using the Odyssey Software program from Li-COR.

Although equal amounts of protein were loaded in each lane, slight variations in the actual loading were compensated by normalizing all cyclin signals to an actin signal on the same membrane.

## Competing interests

The author(s) declare that they have no competing interests.

## Authors' contributions

SC, DVP, DK, and MG-H were primarily involved in the membrane-elution production of cells and the analysis of cell sizes and DNA contents. MP and ML were primarily involved in the western blot analyses. DVP. and MP were involved in the quantitation of the western blots. SC was the primary writer of the paper. All of the authors were involved in developing the ideas contained in this paper, and have read and approved the final manuscript.
